# The PINK1 p.I368N mutation affects protein stability and ubiquitin kinase activity

**DOI:** 10.1186/s13024-017-0174-z

**Published:** 2017-04-24

**Authors:** Maya Ando, Fabienne C. Fiesel, Roman Hudec, Thomas R. Caulfield, Kotaro Ogaki, Paulina Górka-Skoczylas, Dariusz Koziorowski, Andrzej Friedman, Li Chen, Valina L. Dawson, Ted M. Dawson, Guojun Bu, Owen A. Ross, Zbigniew K. Wszolek, Wolfdieter Springer

**Affiliations:** 1 Department of Neuroscience, Mayo Clinic, 4500 San Pablo Road, Jacksonville, FL 32224 USA; 2 Mayo Clinic Graduate School of Biomedical Sciences, Jacksonville, FL 32224 USA; 3 Department of Medical Genetics, Institute of Mother and Child, Warsaw, Poland; 4 Institute of Genetics and Biotechnology, Faculty of Biology, Warsaw University, Warsaw, Poland; 5 Department of Neurology, Faculty of Health Science, Medical University of Warsaw, Warsaw, Poland; 6 Neuroregeneration and Stem Cell Programs, Institute for Cell Engineering, Johns Hopkins University School of Medicine, Baltimore, MD 21205 USA; 7 Solomon H. Snyder Department of Neuroscience, Johns Hopkins University School of Medicine, Baltimore, MD 21205 USA; 8 Adrienne Helis Malvin Medical Research Foundation, New Orleans, LA 70130-2685 USA; 9 Department of Neurology, Johns Hopkins University School of Medicine, Baltimore, MD 21205 USA; 10 Department of Physiology, Johns Hopkins University School of Medicine, Baltimore, MD 21205 USA; 11 Department of Pharmacology and Molecular Sciences, Johns Hopkins University School of Medicine, Baltimore, MD 21205 USA; 12 Department of Neurology, Mayo Clinic, Jacksonville, FL 32224 USA

**Keywords:** Parkinson’s disease, PINK1, PARKIN, Ubiquitin, Mitophagy, Autophagy, PARK2, Mitochondria, Phospho-ubiquitin, E3 ubiquitin ligase

## Abstract

**Background:**

Mutations in *PINK1* and *PARKIN* are the most common causes of recessive early-onset Parkinson’s disease (EOPD). Together, the mitochondrial ubiquitin (Ub) kinase PINK1 and the cytosolic E3 Ub ligase PARKIN direct a complex regulated, sequential mitochondrial quality control. Thereby, damaged mitochondria are identified and targeted to degradation in order to prevent their accumulation and eventually cell death. Homozygous or compound heterozygous loss of either gene function disrupts this protective pathway, though at different steps and by distinct mechanisms. While structure and function of PARKIN variants have been well studied, PINK1 mutations remain poorly characterized, in particular under endogenous conditions. A better understanding of the exact molecular pathogenic mechanisms underlying the pathogenicity is crucial for rational drug design in the future.

**Methods:**

Here, we characterized the pathogenicity of the PINK1 p.I368N mutation on the clinical and genetic as well as on the structural and functional level in patients’ fibroblasts and in cell-based, biochemical assays.

**Results:**

Under endogenous conditions, PINK1 p.I368N is expressed, imported, and N-terminally processed in healthy mitochondria similar to PINK1 wild type (WT). Upon mitochondrial damage, however, full-length PINK1 p.I368N is not sufficiently stabilized on the outer mitochondrial membrane (OMM) resulting in loss of mitochondrial quality control. We found that binding of PINK1 p.I368N to the co-chaperone complex HSP90/CDC37 is reduced and stress-induced interaction with TOM40 of the mitochondrial protein import machinery is abolished. Analysis of a structural PINK1 p.I368N model additionally suggested impairments of Ub kinase activity as the ATP-binding pocket was found deformed and the substrate Ub was slightly misaligned within the active site of the kinase. Functional assays confirmed the lack of Ub kinase activity.

**Conclusions:**

Here we demonstrated that mutant PINK1 p.I368N can not be stabilized on the OMM upon mitochondrial stress and due to conformational changes in the active site does not exert kinase activity towards Ub. In patients’ fibroblasts, biochemical assays and by structural analyses, we unraveled two pathomechanisms that lead to loss of function upon mutation of p.I368N and highlight potential strategies for future drug development.

**Electronic supplementary material:**

The online version of this article (doi:10.1186/s13024-017-0174-z) contains supplementary material, which is available to authorized users.

## Background

Parkinson's disease (PD) is the second most common neurodegenerative disease and is characterized by mitochondrial dysfunctions and loss of dopaminergic neurons [1, 2]. While most cases of PD are sporadic, mutations in the *PTEN-induced putative kinase 1 (PINK1)* and *PARKIN (PARK2)* are the most common causes of early-onset forms of PD [3, 4]. It is known that the mitochondrial kinase PINK1 and the cytosolic E3 ubiquitin (Ub) ligase PARKIN functionally cooperate during mitochondrial quality control to identify, label and remove damaged organelles [5].

In healthy mitochondria, newly translated full-length PINK1 (~63 kDa) is constitutively imported through the TOM complex, the translocase of the outer mitochondrial membrane (OMM) and then through the TIM complex, the translocase of the inner mitochondrial membrane (IMM). Upon import, the N-terminal mitochondrial targeting sequence (MTS) of PINK1 is cleaved off by the matrix processing peptidase (MPP) and the intermediate isoform (~60 kDa) is further processed by the Presenilin-associated rhomboid-like protein (PARL) in the IMM to generate a 52 kDa PINK1 fragment. This cleaved form of PINK1 is exported back to the cytosol by an unknown mechanism and degraded by the Ub/proteasome system (UPS) [6–9].

Upon mitochondrial depolarization, full-length PINK1 is no longer imported into mitochondria, but accumulates, with the kinase domain facing the cytosol, on the OMM and forms a dimeric structure associated with the TOM complex [10–12]. This allows phosphorylation of its substrates, the small modifier protein Ub and its E3 ligase PARKIN, at a conserved Ser65 residue [13–15]. Both PINK1-mediated phosphorylation events fully activate the auto-inhibited enzymatic functions of PARKIN and further facilitate its recruitment from the cytosol [16]. Then, PINK1 and PARKIN together cooperatively label damaged mitochondria in a feed forward mechanism with phosphorylated poly-Ub chains that serve as the “mitophagy tag” for their removal via the autophagy/lysosome system [17–21]. Interference at any of these steps abrogates protection through PINK1/PARKIN-directed mitochondrial quality control.

A deeper understanding of the particular pathomechanisms of individual PARKIN mutations has further allowed delineating the regulation and sequence of events along this process [22]. These detailed structure-function studies have already spurred efforts for a rationalized drug design to activate PARKIN through different strategies [23]. Similarly, missense mutations in PINK1 can interfere with mitochondrial quality control through different molecular mechanisms [24]. For instance PINK1 p.Q456X results in instability of its transcript through non-sense mediated decay, leading to a complete loss-of-function on the protein level [25]. In contrast, the mutant p.G411S is expressed and upon damage forms a dimer on the OMM similar to PINK1 wild type (WT) [26]. However, in addition to partial loss of its kinase activity, in a heterodimer with PINK1 WT, p.G411S also exerts a partial, dominant-negative effect. This lowers overall protection through mitochondrial quality control and enhances risk for PD even in heterozygous carriers.

Towards a complete dissection of PINK1 regulation and a rationalized drug design in the future, we characterized here the novel p.I368N mutation in a combined clinical-genetic and structure-function approach. In patients’ fibroblasts, we found that stabilization of PINK1 p.I368N on the OMM is greatly impaired upon mitochondrial stress. Additional structural analysis revealed changes in the active site of PINK1 p.I368N that should affect ATP and substrate binding. Indeed, in cells we observed loss of kinase activity even under conditions of forced expression. Altogether, our study identified two distinct molecular mechanisms that can drive pathogenicity of PINK1 missense mutations and might help guiding future drug design.

## Methods

### Clinical investigation

The Polish family with the PINK1 p.I368N mutation was recruited to participate in this research study from the movement disorders clinic of the Department of Neurology of the Medical University of Warsaw in Warsaw, Poland. The diagnosis of PD was made based on the UK Parkinson’s Disease Society Brain Bank clinical diagnostic criteria [27]. At the time of blood and skin specimens retrieval, the research subjects were independently clinically examined by two board certified neurologists (DK and ZKW) utilizing standardized narrative Mayo Clinic (MC) history, standardized MC narrative family history, and standardized MC neurological examination [28]. In addition the Hoehn and Yahr and Mini Mental State examination scales were obtained [29, 30]. The blood samples and 3 mm single skin specimens were retrieved in routine fashion by utilizing the standard universal precautions. Both subjects were right handed and parkinsonian features were more expressed on the dominant side. The response to the standard antiparkinsonian therapy including levodopa was excellent and there was no need to proceed with surgical interventions such as for example a deep brain stimulation.

### Genetic examination

Material for analysis of genomic DNA was extracted from blood and fibroblasts using a standard protocol. To confirm the genotype (c.T1103A/c.T1103A; p.I368N/p.I368N) and exclude other mutations in *PINK1*, all coding exons were directly sequenced by Sanger method and analyzed against the reference sequence NM_032409.2 (NCBI36/hg18). As far as we know this is the only family carrying this specific mutation in the Polish population. Though the analyzed siblings both carry a homozygous mutation, there was no consanguineous marriage of their parents. Gene rearrangements were analyzed by multiple ligation-dependent probe amplification (MLPA) technique using available tests SALSA P051 PD probes (MRC Holland) – probes for all *PINK1* exons. Mutations in PINK1 and PARKIN were excluded in control fibroblast cells by sequencing of all coding exons.

### Modeling PINK1 structures and refinement

The modeling of the full-length human PINK1 protein, NP_115785.1 (581 amino acid residues), has been described recently [26]. In brief, each individual domain was modeled as a separate unit and built into a composite full-length structure. The hybrid model is derived from consensus between the programs PRIME (Prime v3.0, Schrödinger, LLC, New York, NY) [31, 32], YASARA SSP/Homology/PSSM Method [31–38], DISTILL (Porter) and TASSER [39–44] and combines homology, threading, *ab initio*, and compositing techniques.

### Modification and mutation modeling

Mutations of amino acids were completed using the Maestro within the Schrödinger suite and parameterized using OPLS2.0 and Amber FF. Additionally, Maestro was used for placing mutated residues (or extending) automatically within an existing peptide chain. Also, MacroModel features within Maestro allow for the quick minimization of the structure for local geometry fixes to correct stereochemistry and packing of the amino acids. Further minimization was completed on the post-Schrödinger model using OPLS2005 within YASARA2, which has an AMBER set and can be used to further parameterize modifications to import into existing molecular dynamics integration engines, as the parameters for the modification are well documented for YASARA and Schrödinger [32, 37, 45–47].

### Prediction of binding energy changes in proteins using FoldX algorithm

For each proposed mutation locus, we equilibrated the corresponding flexibility zone in the wild type complex and evaluated the free energy of unfolding (ΔG_wt_) with the KB potential FoldX [48]. Combined with our own codes, we have a method to scan across trajectories and examine interaction zones of interest. We repeated the calculation for the *mutant* (p.I368N) complex to obtain the respective free energy of unfolding (ΔG_mutant_). The difference between these two quantities is our estimate of the experimental change in binding free energy induced by the mutation [49]: ΔΔG = ΔG_wt_- ΔG_mutant_.

### Molecular dynamics simulation and methods

MDS was completed on each model for conformational sampling, using methods previously described [50–53]. Each PINK1 system was minimized with relaxed restraints using Steepest Descent and Conjugate Gradient PR, and equilibrated in solvent with physiological salt conditions, as described in the literature [50–53]. After equilibration was established, each system was allowed to run an additional MD production length of >250 nanoseconds. The primary purpose of MD for this study was conformational stability, refinement, and interaction calculations that may occur at the active site or dimer interface. The protocol for refinement include the following steps: (1) Minimization with explicit water molecules and ions, (2) Energy minimization of the entire system, and (3) MDS for >2 ns to relax to the force field (AMBER03). Following the refinement protocol, production simulations were completed to collect data.

### Molecular dynamics protocol

AMBER03 force fields were used with the current release of NAnoscale Molecular Dynamics 2 engine [54, 55]. The PINK1 systems simulated, including hydrogens, consist of 1.74 × 10^4^ atoms prior to solvation with TIP3P water and ions. In all cases, we neutralized with counter-ions, and then created a solvent with 150 mM Na + Cl- to recreate physiological strength. TIP3P water molecules were added around the protein at a depth of 12–15 Å from the edge of the molecule depending upon the side [56]. Our protocol has been previously described in the literature [50, 51, 53, 57]. Solvated protein simulations consist of a box with 2.23–3.35 × 10^5^ atoms including nucleic acids, proteins, counter-ions, solvent ions, and solvent waters. Simulations were carried out using the particle mesh Ewald technique with repeating boundary conditions with a 9 Å nonbonded cut-off, using SHAKE with a 2-fs timestep. Pre-equilibration was started with three stages of minimization with 10,000 steps of SD, PRCG, relaxing restraints, then followed by 1000 ps of heating under MD, with the atomic positions of nucleic and protein fixed. Then, two cycles of minimization (5000 steps each) and heating (1000–5000 ps) were carried out with soft restraints of 10 and 5 kcal/(mol · Å^2^) applied to all nucleic and protein backbone atoms. Next, 5000-steps of minimization were performed with solute restraints reduced to 1 kcal/(mol · Å^2^). Following that, 2000–4000 ps of MDS were completed using relaxing restraints (1 kcal/(mol · Å^2^)) until all atoms are unrestrained, while the system was slowly heated from 1 to 310 K using velocity rescaling upon reaching the desired 310 K during this equilibration phase. Additionally, NPT equilibration based MD was used with velocity rescaling for 1–2 ns. Finally, production runs of MD were carried out with constant pressure boundary conditions (relaxation time of 1.0 ps). A constant temperature of 310 K was maintained using the Berendsen weak-coupling algorithm with a time constant of 1.0 ps. SHAKE constraints were applied to all hydrogens to eliminate X-H vibrations, which yielded a longer simulation time step (2 fs). Our methods for equilibration and production run protocols are in the literature [50, 51, 53, 57]. Equilibration was determined from a global flattening of RMSD over time after an interval of 1–3 ns or similarly with total energy. Translational and rotational center-of-mass motions were initially removed. Periodically, simulations were interrupted to have the center-of-mass removed again by a subtraction of velocities to account for the “flying ice-cube” effect [58]. Following the simulation, the individual frames were superposed back to the origin, to remove rotation and translation effects. Archived snapshots were recorded every 2 ps, yielding >2000 snapshots per simulation.

### Docking of kinase PINK1 and substrate ubiquitin

Using mixture of Piper protein-protein docking for generating 40,000 conformers of random seed orientation over the PINK1 and ubiquitin proteins, we applied restriction parameters as follows: only kinase domains from PINK1 (N-lobe and C-lobe) and Ser65 face of Ub were weighted with increased attraction parameters +0.25, whilst we also did multiple LC-MOD/MC generated side chain re-orientations during the final combination with an simulated annealing protocol to allow for settling of interactions before applying a PRCG minimization protocol. We examined the docking interfaces with brief bursts of simulated annealing and MDS for further refinement for taking the top seeded structures and elimination of the poor scorers. Top surviving poses were scored and best from each series was retained [50, 52, 53, 59, 60]. The best pairs generated were refined and studied using MDS calculations.

### Cell culture

Primary human dermal fibroblasts were grown in Dulbecco’s modified Eagle medium (DMEM, Invitrogen) supplemented with 10% fetal bovine serum (FBS, BioWest), 1% PenStrep and 1% non-essential amino acids (both Invitrogen). Control fibroblasts were received from Cell Applications Inc., mutations in PINK1 and PARKIN genes were excluded by sequencing and PINK1-dependent responses in those cells have been characterized before [18, 25, 26]. HeLa cells (ATCC) were cultured in DMEM supplemented with 10% FBS. Stable HeLa cell clones expressing EGFP-Parkin or 3xFLAG-Parkin C431S have been described before [61]. All cells were maintained at 37 °C, 5% CO_2_ in a humidified atmosphere.

### Chemical treatments, siRNA and DNA transfection of cells

Carbonyl cyanide m-chlorophenyl hydrazone (CCCP), epoxomicin and cycloheximide were purchased from Sigma-Aldrich, valinomycin from Axxora. siRNA transfections were performed with 20nM control (all stars negative control) or PINK1-specific siRNA (5′ GACGCTGTTCCTCGTTATGAA-3′) using HiPerfect (all from Qiagen) or Lipofectamine2000 (Invitrogen). siRNA resistant PINK1-V5 constructs have been described before [5, 24]. The mutations p.F104A, p.L347P and p.I368N were introduced by site-directed mutagenesis and verified by Sanger sequencing. The standard transfection protocol for DNA was as follows: for one well of a 12-well plate 1 μg of DNA and 2.5 μl of Lipofectamine2000 (Invitrogen) were each mixed with 100 μl of Opti-MEM media (Invitrogen), incubated for 5 min at room temperature, mixed together and incubated 20 min before adding to the cells. For low-level expression DNA and lipofectamine amounts were reduced by 50–75% (total DNA per 12 well: 0.5 or 0.25 μg).

### RNA extraction and real-time PCR analysis

Total mRNA from fibroblasts was prepared using the RNeasy mini kit (Qiagen) according to the manufacturer’s protocol and then reverse-transcribed into cDNA using a High Fidelity cDNA kit (Roche Applied Science). cDNA was amplified by real-time PCR on a LightCycler 480 system (Roche) using Taqman probes (BioRad PINK1 Id qHsaCEP0053094 and RPL27 Id qHsaCEP0051648). Levels were determined using the second derivative method.

### Antibodies

The following primary antibodies were used for Western blot (WB) and/or immunofluorescence (IF): rabbit anti-CDC37 (WB, 1:1,000, 4793, Cell Signaling), mouse anti-HSP90 (WB, 1:2,000, 610419, BD), mouse anti-FLAG (WB, 1:150,000, F3165, Sigma Aldrich), mouse anti-GAPDH (WB, 1:150,000, H86504M, Meridian Life science), mouse anti-Mitofusin 1 (WB, 1:5,000, ab57602, Abcam), rabbit anti-p38 MAPK (WB, 1:1,000, #9212, Cell Signaling), rabbit anti-PINK1 (WB, 1:2,000, BC100-494, Novus Biologicals), rabbit anti-PINK1 (WB, 1:2,000, IF, 1:100, #6946, Cell Signaling), mouse anti-Parkin (WB, 1:3,000, #4211, Prk8, Cell Signaling), mouse anti-TOM20 (IF, 1:250, sc-17764, Santa Cruz Biotechnology), rabbit anti-TOM20 (WB, 1:20,000, 11802-1-AP, ProteinTech Group), rabbit anti-TOM40 (WB, 1:1,000, 18409-1-AP, ProteinTech Group), mouse anti-Ub (WB, 1:1,000, MAB1510, ubi-1, Millipore), rabbit anti-p-Ser65-Ub (WB, 1:5,000-1:15,000, IF, 1:250, in house [18]), mouse anti-V5 (WB, 1:5,000, R960-25, Invitrogen), rabbit anti-V5 (WB, 1:5,000, ab9115, Abcam), mouse anti-vinculin (WB, 1:100,000-500,000, V9131, Sigma). Streptavidin HRP (WB, 1:150,0000, Pierce).

### Protein extraction, SDS-PAGE and Western blot (WB)

Cells were collected and lysed in RIPA buffer (50 mM Tris pH 8.0, 150 mM NaCl, 1% NP-40, 0.5% deoxycholate, 0.1% SDS) plus Complete protease and PhosSTOP phosphatase inhibitors (Roche Applied Science) on ice for 30 min. Proteins were collected as supernatants after centrifugation at 20,000 g for 15 min at 4 °C. SDS–PAGE was performed using 8–16% or 16% Tris Glycine gels (Invitrogen). Proteins were transferred onto PVDF membranes and detected using standard immunoblotting procedures. To detect phosphorylated PINK1, 8% Tris Glycine gels containing 50 μM Phos-tag acrylamide (Wako chemicals) and 100 μM ZnCl_2_ were used [18]. After electrophoresis, Phos-tag acrylamide gels were washed using transfer buffer with 0.01% SDS and 1 mM EDTA for 20 min and then with transfer buffer containing 0.01% SDS without EDTA for 10 min to remove excess Zn^2+^ before transfer.

### Subcellular fractionation and solubility analysis

Cells were harvested in fractionation buffer containing 20 mM HEPES-KOH (pH 7.4), 1 mM EDTA, 250 mM sucrose and cocktail of protease and phosphatase inhibitors (Roche Applied Science). After homogenization with a 22 G syringe (20 strokes), nuclei and cell debris were removed by centrifugation at 1500 g for 5 min. The post-nuclear supernatant (PNS) was transferred into a new tube and centrifuged at 12,000 g for 10 min. The mitochondria-enriched pellet (‘mitochondrial fraction’) was dissolved in RIPA buffer. Cytoplasmic fractions were isolated as supernatant after ultracentrifugation at 100,000 g for 30 min. For solubility studies, the PNS was dissolved in RIPA buffer and pelleted at 14,000 g for 15 min. The supernatant was used as the “soluble” fraction, the pellet was further washed and solubilized in 8 M urea and used as the “insoluble” fraction.

### Analysis of ubiquitin charging of PARKIN C431S

Transfected HeLa 3xFLAG-Parkin C431S cells [61] were harvested in preheated (95 °C) SDS lysis buffer (50 mM Tris pH 7.6, 150 mM NaCl, 1% SDS). Lysates were homogenized by 10 strokes through a 20G needle. To verify the band shift by oxyester formation, aliquots of lysates were treated with or without NaOH (final concentration: 100 mM) for 1 h at 37 °C before loading onto SDS gels.

### Immunofluorescence (IF)

Fibroblasts were seeded onto PDL (P0899, Sigma-Aldrich) coated glass coverslips treated with valinomycin, CCCP or left untreated. Cells were then fixed with 4% (w/v) paraformaldehyde, permeabilized with 1% Triton-X-100, blocked with 10% goat serum and incubated with primary antibodies followed by secondary antibodies (anti-rabbit Alexa Fluor-488, anti-mouse IgG Alexa Fluor-568, 1:1000, Invitrogen). Nuclei were stained with Hoechst 33342 (1:5000, Invitrogen). For staining of PINK1 tyramide signal amplification (T20922, Invitrogen) was used. Coverslips were mounted onto microscope slides using fluorescence mounting medium (Dako). High resolution confocal images were taken with an AxioObserver microscope equipped with an ApoTome Imaging System (Zeiss).

### High content imaging (HCI)

To quantify p-Ser65-Ub levels in valinomycin treated fibroblasts and EGFP-PARKIN translocation in CCCP treated HeLa cells, we used the BD Pathway 855 HCI platform (BD Biosciences) as described earlier [18, 26, 61]. To collect signal only from cells transfected with siRNA and various PINK1-V5 constructs, co-expressed mCherry fluorescent intensity was used as a restriction parameter for the analysis of PARKIN translocation.

### (Co-)Immunoprecipitation

HeLa cells were transiently transfected with V5-6xHis-tagged PINK1 WT, p.I368N or p.L347P for 48 h and were then treated with 15 μM CCCP for 2 h or left untreated. Cells were lysed in co-IP buffer (50 mM HEPES pH 7.5, 150 mM NaCl, 10 mM KCl, 1.5 mM MgCl_2_, 1 mM EDTA, 0.5 mM EGTA, 10% glycerol and 0.2% NP-40) supplemented with protease and phosphatase inhibitors (Roche Applied Science) and briefly sonicated. For PINK1-V5 immunoprecipitation (IP), total cell lysate was incubated overnight at 4 °C with mouse anti-V5-agarose beads (A7345, Sigma-Aldrich). Formed immuno-complexes were spun down, washed 3x with co-IP buffer and eluted from beads with 50 μl 2x SDS sample buffer and boiled at 95 °C. 10 μg of input cell lysates and 10 μl of immunoprecipitates were analyzed by Western blot.

### *In vitro* kinase assay

Immunoprecipitated WT and p.I368N PINK1-V5 from CCCP treated and untreated HeLa cells were washed twice with co-IP buffer and once with *in vitro* phosphorylation buffer (20 mM HEPES pH 7.4, 50 mM NaCl, 5 mM MgCl_2_, 0.1 mM EGTA, 0.01% Triton X-100). After complete removal of the buffer 100 μl of the *in vitro* reaction mixture was added. The reaction contained 1 μg of N-terminally biotinylated Ub (Boston Biochem), 1 mM TCEP (Gold Bio) and 2 mM ATP trisodium salt (Sigma-Aldrich) in phosphorylation buffer. Reactions were carried out at 37 °C under constant agitation for 24 h, stopped by addition of 6x SDS sample buffer, heated to 56 °C for 15 min and analyzed by immunoblotting using p-Ser65-Ub antibody. Detection of biotinylated Ub with streptavidin-HRP (Pierce) served as loading control.

### Densitometry, PINK1 half-life calculation and statistical methods

Densitometric analysis of Western blots was performed with the Image Studio software (LiCor). The half-life of PINK1 proteins was calculated by non-linear one phase decay curve fitting using GraphPad Prism version 6.

All quantitative results are expressed as mean ± SEM from at least three independent experiments. Statistical comparisons were performed using one-way and two-way ANOVA, respectively, with Tukey’s post-hoc test (*, *p* < 0.05; **, *p* < 0.005; ***, *p* < 0.0005). All analyses were performed using GraphPad Prism version 6.

## Results

### Clinical features of PINK1 p.I368N are similar to sporadic late-onset PD

Here we analyzed two patients that are homozygous carriers of a recently identified *PINK1* c.T1103A (p.I368N) mutation [62]. Detailed clinical data of the affected members are provided in Additional file 1: Table S1. Both patients have early-onset and slowly progressive PD that started at relatively young age (28 and 33 years, respectively), consistent with the typical clinical presentation of homozygous PINK1 loss-of-function [63]. Some clinical features (e.g. laterality of parkinsonism, resting tremor, bradykinesia and good response to levodopa) are indistinguishable from sporadic late-onset PD. Yet, REM sleep behavior disorder (RBD), which is a clinical biomarker of synucleinopathies, was not detected in these patients [64]. Both patients show mild autonomic failure (e.g. constipation), but no dementia, and no depression or anxiety, which are occasionally recognized as a non-motor symptom of PINK1-associated PD [65].

### Structural changes of the p.I368N mutation distort the ATP-binding site of PINK1

**Fig. 1:**
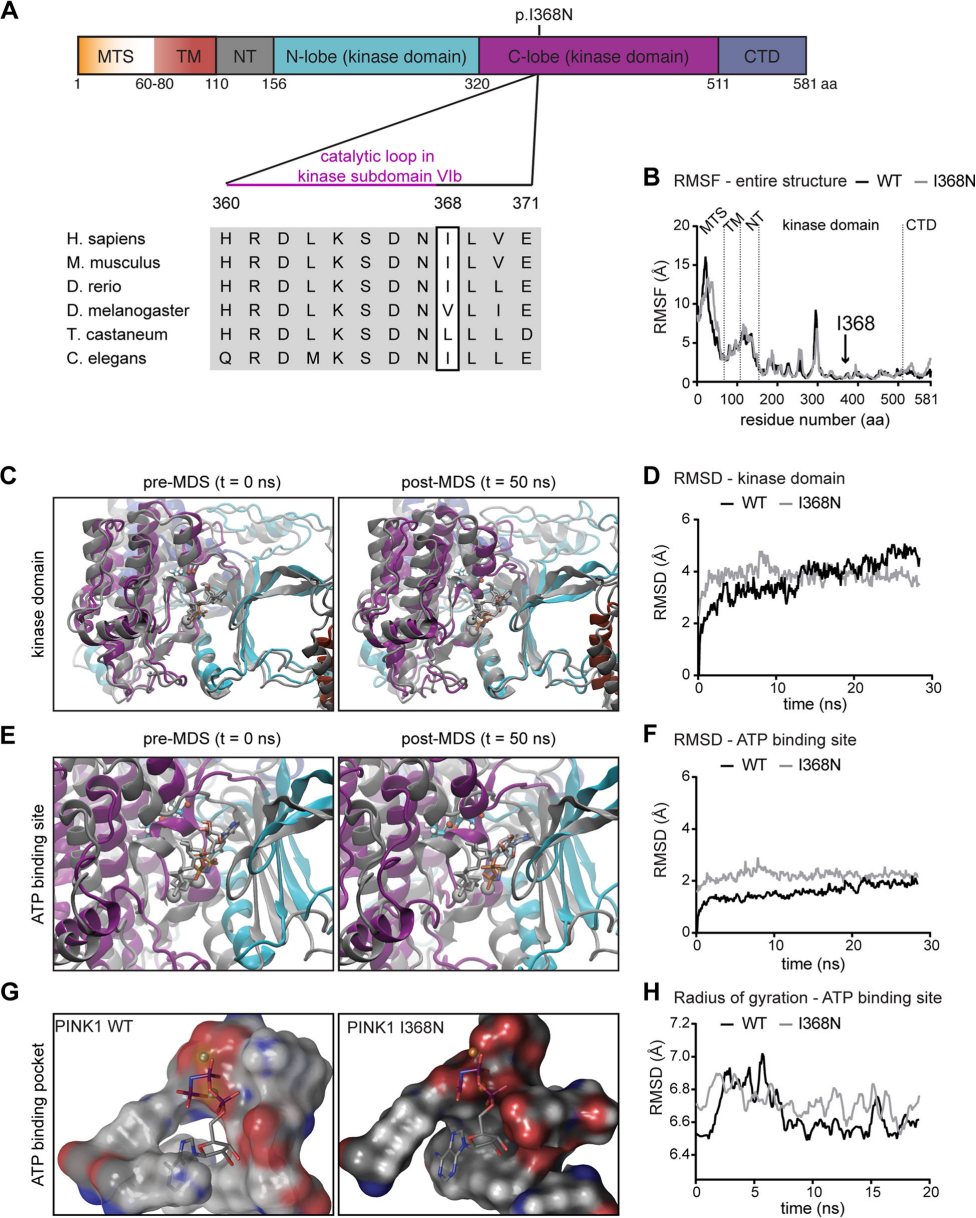
The PINK1 p.I368N mutation is positioned in the kinase domain and alters the ATP-binding site. **a** Domain structure of human PINK1 protein. Individual domains are color-coded and labeled: mitochondrial targeting sequence (MTS); transmembrane domain (TM); N-terminal, regulatory domain (NT); N-lobe and C-lobe of the kinase domain; C-terminal domain (CTD). Residues surrounding Ile368 were aligned with corresponding sequences of PINK1 orthologs from different species. Horizontal bar denotes the catalytic loop in kinase subdomain VIb. **b** Root mean square fluctuations (RMSF) of the entire PINK1 structures. Dotted lines separate individual domains of PINK1 that include MTS, TM, NT, kinase domain and CTD. An arrow highlights the position of the p.I368N mutation. **c** Superposition overlay of the kinase domains before and after 50 ns of MDS. Structures are shown in cartoon ribbons with PINK1 WT in gray and p.I368N mutant in color according to the domain structure in (**a**). Residues Ile368 and Asn368 are shown in licorice rendering using gray or standard atom coloring, respectively. The Mg^2+^ ions are gray colored Van der Waals (VdW) spheres for both and the ATP-analogs in the active sites are shown in gray for WT and Goodsell coloring for p.I368N. **d** Root mean square deviation (RMSD) of the kinase domain of PINK1 WT and p.I368N mutant, shows only minor structural differences. **e** Zoom into the ATP-binding sites of PINK1 WT and p.I368N mutant in superposition overlay before and after 50 ns of MDS as described for (**c**). **f** RMSD of the ATP-binding pocket in the kinase domains of WT and mutant PINK1. Pocket residues include positions: 161,163, 217, 218, 219, 237, 240, 275, 316, 318, 319, 320, 321, 322, 325, 326, 327, 362, 363, 365, 366, 367, 368, 369, 383, 384, 385, 386, and 387. **g** The surface of the ATP-binding pocket of WT and p.I368N mutant PINK1 is shown for comparison in standard atom coloring. The Mg^2+^ ion (VdW sphere) and the ATP-analog (sticks colored by atom type) in the active site are given. **h** Radius of gyration measurements on the ATP-binding pocket from WT and mutant PINK1 provides the extent to which an object is extended from its center of mass


The residue Ile368 is located at the +1 position of the catalytic loop of the consensus motif HRDLKxxN in Ser/Thr kinases (Fig. 1a). Using homology modeling, we have recently generated a structural model of full-length PINK1 at an all-atom resolution [26]. To examine how the p.I368N mutation would affect protein structure we performed molecular modeling and free, unbiased molecular dynamics simulations (MDS) (Additional file 2: Movie S1 and Additional file 3: Movie S2 for PINK1 WT and p.I368N mutant, respectively). The calculated Root Mean Square Fluctuations (RMSF) provide the movement of each individual residue of PINK1 (581 amino acids) as a time-averaged sum over the course of the simulation. Though global conformational changes were not observed, overall RMSF of PINK1 p.I368N were slightly lower than WT, yet with few higher peaks around residues of the ATP-binding pocket (Fig. 1b). Overlay of the WT and mutant kinase domains together with Root Mean Square Deviations (RMSD), as a measure of structural change, revealed an immediate but minor difference before and after MDS (Fig. 1c, d). However, specifically the orientation of helices surrounding the ATP-binding pocket was altered in the mutant compared to PINK1 WT (Fig. 1e) and resulted in local changes of RMSD within the ATP-binding site (Fig. 1f). In addition, surface analyses revealed a distorted ATP pocket shape for p.I368N (Fig. 1g). Together with FoldX measurements of Gibb’s free energy that indicated decreased local stability (Additional file 4: Table S2) and an increase in the radius of gyration (Fig. 1h) this indicated a negative effect on the catalytic activity of PINK1.

Additional file 2: **Movie S1.** MDS of PINK1 WT. A zoom into the kinase domain of PINK1 WT is shown for 50 ns simulation. PINK1 structure is depicted in cartoon ribbons colored according to the domain structure. Residue Ile368 is highlighted in licorice rendering using standard atom coloring. The Mg2+ ion is a gray colored Van der Waals (VdW) sphere and the ATP-analog in the active sites is shown in Goodsell coloring. There is no significant difference between either of the monomeric halves of the PINK1 WT dimer during the MDS. (MP4 3019 kb)



Additional file 3: **Movie S2.** MDS of PINK1 p.I368N. A zoom into the kinase domain of PINK1 p.I368N is shown for 50 ns simulation. PINK1 structure is depicted in cartoon ribbons colored according to the domain structure. Residue Asn368 is highlighted in licorice rendering using standard atom coloring. The Mg2+ ion is a gray colored Van der Waals (VdW) sphere and the ATP-analog in the active sites is shown in Goodsell coloring. There is no significant difference between either of the monomeric halves of the PINK1 mutant dimer during the MDS.  (MP4 7144 kb)


### PINK1 p.I368N decreases binding efficiency and mispositions the substrate Ub

**Fig. 2:**
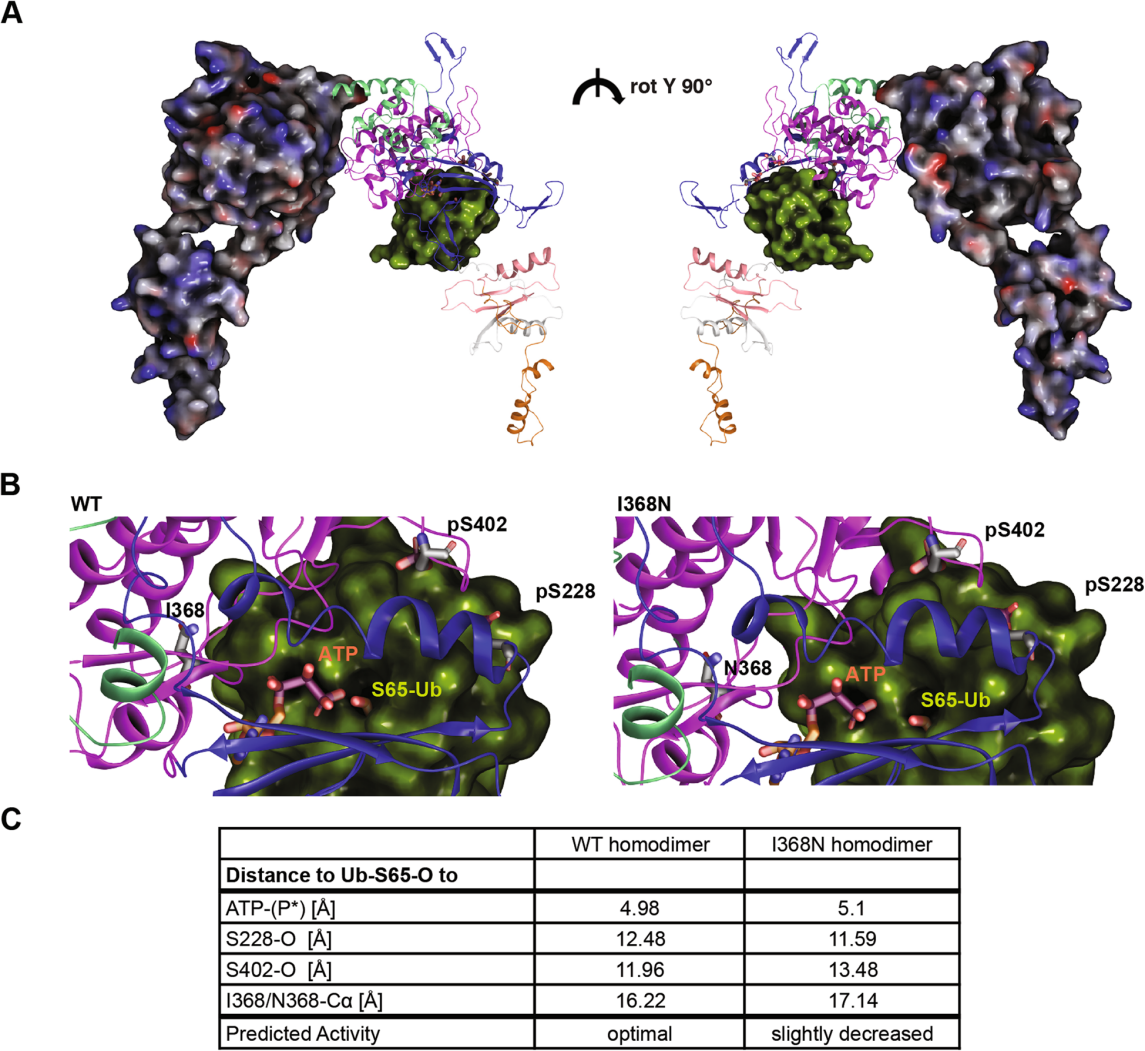
Protein docking of the kinase PINK1 and the substrate Ub. **a** Structural model of a PINK1 p.I368N homodimer docked with Ub in the kinase domain (between the N- and C-lobe). One molecule of the PINK1 dimer is shown in electrostatic surface rendering. The other one is shown in ribbons colored according to the individual domains of PINK1. The p-Ser228, p-Ser402, and Asn368 residues are shown in licorice sticks with the carbons colored in gray. ATP is shown in licorice rendering with orange carbons. The substrate Ub is shown in yellow-green surface culled to reveal Ser65 residue in VdW. Both sides of the dimer are rotated 180°. **b** Zoom into the active site region. Left panel: PINK1 WT (residues Ile368, p-Ser228, and p-Ser402 are highlighted) docked with ATP (terminal phosphate is highlighted), and Ub (Ser-65 is highlighted). Right panel: PINK1 p.I368N mutant with the same residues highlighted for comparison. **c** Metric for enzymatic comparison of the of PINK1 WT and p.I368N mutant is given in the table. Atomic distances between the docked Ub (Ser65-oxygen), the terminal phosphate of ATP (P*), and PINK1 residues (p-Ser228-oxygen, p-Ser402-oxygen, I368-Calpha or N368-Calpha) were determined

To study the enzymatic activity, we had recently docked the substrate Ub into the kinase domain of PINK1 WT [26]. We could show that auto-phosphorylation of PINK1 at Ser228 and Ser402 increased interactions with Ub that not only stabilized the kinase/substrate complex but also positively affected atomic distances for a more efficient phosphate transfer. Here, we docked Ub to a p.I368N homodimer to investigate binding and alignment of the substrate within the active site of the kinase (Fig. 2a). Indeed, closer inspection of the mutant enzymatic complex, revealed a slight mispositioning of the Ub substrate (Fig. 2b). However, distances measured between key atoms of PINK1, ATP (terminal phosphate), and Ub (oxygen of Ser65) were only slightly altered, and still within the range for effective catalysis. In summary, structural analyses suggested a kinase defect for the PINK1 p.I368N mutation would likely be caused by impaired ATP stabilization in the active site and potentially altered binding of the substrate Ub.

### PINK1 p.I368N mutant protein is not properly stabilized upon dissipation of the mitochondrial membrane potential

**Fig. 3:**
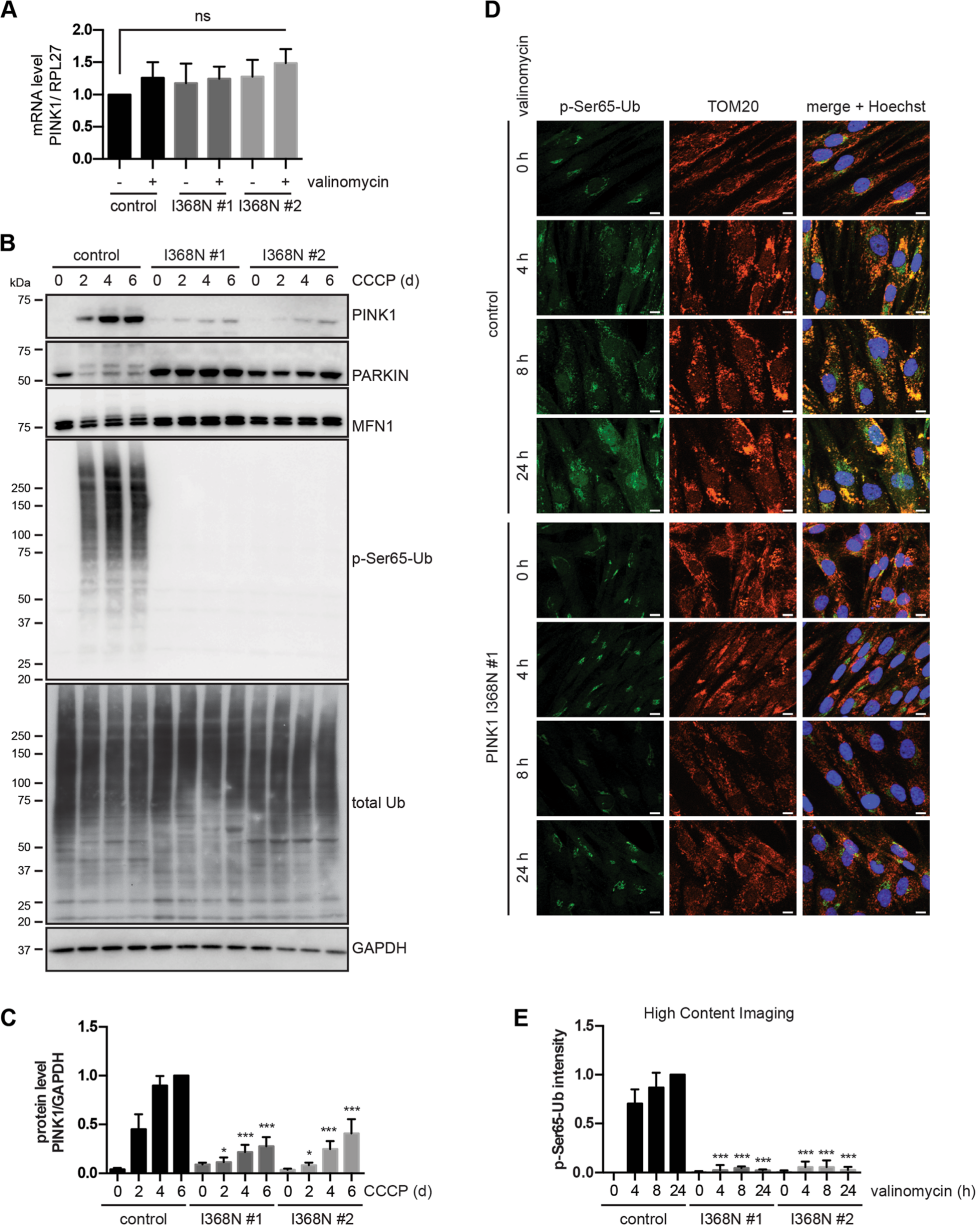
Full-length PINK1 protein and kinase activity are decreased in p.I368N mutant fibroblasts. **a** qRT-PCR was used to measure mRNA levels of PINK1 and housekeeping gene RPL27 in untreated cells and cells treated with 1 μM valinomycin for 24 h. Values of the PINK1/RPL27 mRNA ratio were normalized to untreated control cells. Error bars indicate mean ± SEM from six independent experiments (one-way ANOVA with Tukey’s post hoc; ns, not significant). **b** WT control and two PINK1 p.I368N fibroblasts were treated with 10 μM CCCP for 0, 2, 4 or 6 days and total lysates were analyzed by WB with the indicated antibodies. GAPDH served as a loading control. Slower migrating PINK1, PARKIN, and Mitofusin1 (MFN1) protein species indicate activated and/or modified forms and were only observed in control cells. p-Ser65-Ub signal increased in control cells over time but was undetectable in PINK1 p.I368N mutant cells. **c** Densitometric analysis of full-length PINK1 protein from (**b**). Values were normalized to controls cells treated for 6 days with CCCP. Error bars indicate mean ± SEM from four independent experiments. Values were tested for statistical significance compared to control cells (two-way ANOVA with Tukey’s posthoc test, *, *p* < 0.05, ***, *p* < 0.0005). **d** Representative images of fibroblasts upon treatment with 1 μM valinomycin. Cells were stained with anti p-Ser65-Ub (green) and TOM20 (red). Nuclei are shown in blue (Hoechst). Scale bars indicate 10 μm. Upon treatment, p-Ser65-Ub is induced only in control, but not in PINK1 p.I368N fibroblasts. **e** p-Ser65-Ub signal upon valinomycin treatment was quantified by HCI. Control and PINK1 p.I368N fibroblasts were incubated with 1 μM valinomycin for 0, 4, 8 and 24 h. Cells were fixed and stained with p-Ser65-Ub antibodies and a nuclear counterstain (Hoechst). Mean intensity of p-Ser65-Ub in a cytoplasmic ring around the nucleus was measured and normalized to values of untreated and 24 h valinomycin treated control cells. Error bars indicate mean ± SEM from three independent experiments (two-way ANOVA with Tukey’s post hoc; ***, *p* < 0.0005)

To study the effects of endogenous mutant PINK1 in cells, we analyzed primary skin fibroblasts from controls and both patients carrying the homozygous p.I368N mutation. We quantified PINK1 mRNA levels before and after mitochondrial depolarization with the K^+^ionophore valinomycin by quantitative reverse transcriptase PCR (qRT-PCR), but found no significant difference between WT and mutant PINK1 fibroblasts (Fig. 3a). Next, we examined PINK1 protein levels under basal conditions and upon mitochondrial damage (Fig. 3b). In the absence of stress, PINK1 protein was hardly detectable by Western blot (WB) under endogenous conditions. While full-length PINK1 WT robustly accumulated in controls upon treatment with the mitochondrial uncoupler CCCP, stabilization of p.I368N mutant PINK1 protein was significantly impaired (Fig. 3b, c). To confirm these findings, fibroblasts were subjected to immunofluorescence (IF) with antibodies against PINK1 and the mitochondrial marker protein TOM20. Consistently, PINK1 signal was considerably weaker in p.I368N mutant cells compared to WT controls (Additional file 5: Figure S1A). Despite significantly reduced protein levels, localization of the mutant PINK1 p.I368N to damaged mitochondria seemed unaltered. This was further corroborated by subcellular fractionations, where both WT and mutant full-length PINK1 were detected in the mitochondrial fraction upon depolarization (Additional file 5: Figure S1B). Taken together, p.I368N mutant cells showed no changes in mRNAs levels, but significantly reduced levels of the full-length PINK1 protein on mitochondria upon depolarization.

### The PINK1 p.I368N mutation affects kinase activity and PARKIN activation

To determine the enzymatic activity of the PINK1 p.I368N mutation under endogenous conditions we used recently developed antibodies specific to the PINK1-phosphorylated form of Ub (p-Ser65-Ub) [18, 19, 26]. While Ub was strongly and time-dependently phosphorylated upon CCCP treatment in control cells, no p-Ser65-Ub signal was detected by WB in cells from patient’s carrying the PINK1 p.I368N mutation (Fig. 3b). The same results although with an overall faster stress response were obtained upon depolarization of mitochondria with valinomycin (Additional file 5: Figure S1C). To further confirm this by an independent method, fibroblasts were treated with valinomycin and analyzed by IF (Fig. 3d). In line, PINK1 mutant cells showed no induction of specific p-Ser65-Ub signal above background, which was quantified by high content imaging (HCI) (Fig. 3e and Additional file 5: Figure S1D). Consistent with the lack of p-Ser65-Ub signal in patients’ cells, PARKIN activation/modification and Mitofusin1 (MFN1) ubiquitylation were not observed (see Fig. 3b). Overall, these results show that the PINK1 p.I368N mutation reduces protein stabilization upon stress resulting in failure to activate PARKIN and mitochondrial quality control.

### Cleaved forms of PINK1 p.I368N protein are stabilized upon proteasome inhibition

**Fig. 4:**
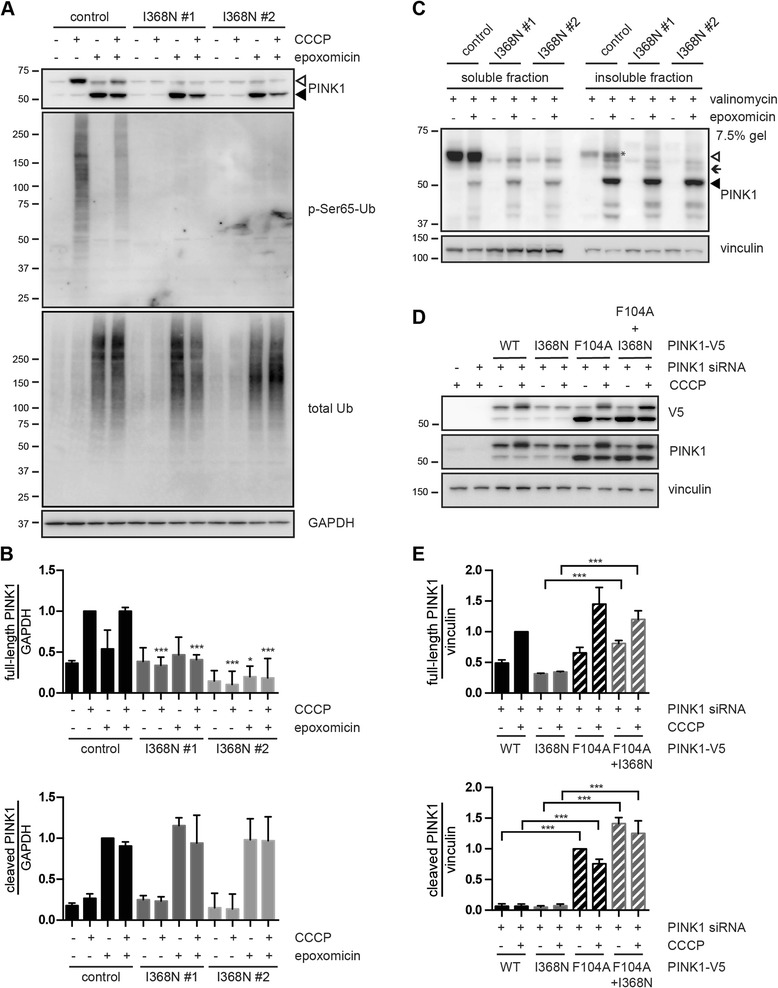
PARL-cleaved forms of PINK1 WT and p.I368N are similarly stabilized upon proteasome inhibition. **a** Control and PINK1 p.I368N fibroblasts were pretreated with 500 nM epoxomicin for 1 h followed by 10 μM CCCP treatment for additional 24 h, as indicated. Only PARL-cleaved PINK1 p.I368N (~52 kDa, black arrowhead), but not the full-length form (~63 kDa, white arrowhead) accumulated with epoxomicin ± CCCP in mutant fibroblasts. Anti-p-Ser65-Ub and total Ub antibodies were used as controls for CCCP and epoxomicin treatment, respectively. GAPDH served as a loading control. **b** WB quantification of full-length (top) and PARL-cleaved (bottom) PINK1 species from control and p.I368N mutant fibroblasts as performed in (**a**). Values of the full-length PINK1/GAPDH ratio were normalized to control cells treated only with CCCP. Values of the cleaved PINK1/GAPDH ratios were normalized to control cells treated only with epoxomicin. Error bars indicate mean ± SEM from three independent experiments (two-way ANOVA with Tukey’s post hoc; *, *p* < 0.05; ***, *p* < 0.0005). **c** Fibroblasts were pretreated with 500 nM epoxomicin for 1 h followed by 1 μM valinomycin treatment for additional 24 h, as indicated. Cells were homogenized in fractionation buffer and proteins were sequentially extracted into soluble (RIPA) and insoluble (urea) fractions and were analyzed by WB using PINK1 antibody. Vinculin served as a loading control. White and black arrowheads indicate PINK1 full-length (mostly in soluble fraction) and the PARL-cleaved form (mostly in insoluble fraction), respectively. Black arrow indicates an intermediate (probably MPP-cleaved) form of PINK1 (~60 kDa), which could be detected in the “insoluble fraction” after epoxomicin treatment. **d** HeLa cells were transfected with control or PINK1 siRNA together with constructs encoding for PINK1-V5 (WT, p.I368N, p.F104A, or the double mutant p.F104A + p.I368N). Cells were left untreated or treated with 10 μM CCCP for 2 h. Levels of full-length and cleaved PINK1 were analyzed by WB using anti-V5 and anti-PINK1 antibodies. Vinculin served as a loading control. **e** Densitometric analysis of the full-length (top) and PARL-cleaved (bottom) PINK1 species from (**d**). Values of the full-length PINK1-V5/vinculin ratio were normalized to CCCP-treated cells expressing WT PINK1-V5. Values of the cleaved PINK1-V5/vinculin ratio were normalized to untreated cells expressing PINK1-V5 p.F104A mutant. Error bars indicate mean ± SEM from three independent experiments (two-way ANOVA with Tukey’s post hoc; ***, *p* < 0.0005)

In order to reveal whether differences in PINK1 protein levels are caused by aberrant processing or protein instability, we co-treated cells with a mitochondrial uncoupler and a proteasome inhibitor to stabilize the cleaved forms of PINK1. WB analysis showed that although full-length PINK1 protein (~63 kDa) was significantly reduced in p.I368N mutant cells upon mitochondrial depolarization, the PARL-cleaved form (~52 kDa) was stabilized upon epoxomicin treatment to the same extent as PINK1 WT in the control cells (Fig. 4a, b). Full-length PINK1 and in particularly the PARL-cleaved form has been reported before as an insoluble protein [7, 66, 67]. Thus, we separately analyzed soluble and insoluble fractions of control and p.I368N mutant fibroblasts that had been treated with a combination of valinomycin and epoxomicin. Full-length WT PINK1 was mostly found in the soluble fraction, while its cleaved form strongly accumulated in the insoluble fraction upon proteasome inhibition (Fig. 4c). While the PARL-cleaved form of p.I368N accumulated in the insoluble fraction similar to PINK1 WT, full-length mutant PINK1 remained barely detectable in either fraction. Yet, we could not identify any distinct, additional cleavage product for p.I368N that would indicate aberrant processing of mutant PINK1. Accordingly, several computational prediction tools failed to report de novo protease cleavage sites for PINK1 p.I368N (data not shown).

### Protein instability of PINK1 p.I368N is masked upon overexpression

To examine whether full-length PINK1 p.I368N is indeed an inherently unstable protein, we compared WT and mutant levels upon overexpression in HeLa cells. In contrast to endogenous PINK1 in fibroblasts, expression levels of full-length and cleaved p.I368N mutant were surprisingly similar to PINK1 WT upon standard transfection (1 μg DNA per well of a 12-well plate), regardless of mitochondrial depolarization (Additional file 6: Figure S2A). However, transfection of only half of the DNA amounts (0.5 μg versus 1 μg), resulted in decreased levels of PINK1 p.I368N compared to WT protein comparable to the endogenous condition in patients’ fibroblasts. In line with previous studies that had raised caution, our data show that high-level PINK1 overexpression can override important regulatory mechanisms and may mask defects of PINK1 mutations [26].

Although the exact reasons are unclear, epoxomicin treatment negatively affected stabilization of full-length PINK1 on damaged mitochondria in line with the literature [68]. To prevent any confounding effect of proteasome inhibition, we made use of an engineered mutation that specifically blocks degradation of cleaved PINK1. It is known that PINK1, once imported through the IMM, is cleaved by PARL in its transmembrane region between Ala103 and Phe104, resulting in its degradation by the N-end rule pathway [7]. The F104A mutation is able to block degradation of the PARL- cleaved form of PINK1 independently of the proteasome activity. Upon knockdown of endogenous PINK1 in HeLa cells, we expressed PINK1 siRNA-resistant variants with single or double p.I386N and p.F104A mutations at low levels (Fig. 4d). Under these conditions, full-length PINK1 p.I368N was less stable than PINK1 WT, but similar levels were reached for the p.F104A mutant. Cleaved PINK1 was barely detectable for WT or p.I368N, but strongly accumulated in the single and double mutants harboring the p.F104A mutation. Strikingly, also full-length PINK1 p.I368N was significantly stabilized upon CCCP treatment by the presence of the p.F104A mutation (Fig. 4e). Altogether these results show that while generation, processing and stability of PINK1 p.I368N are not impaired under basal conditions, specifically the mutant full-length form does not properly accumulate on the OMM upon mitochondrial stress.

### PINK1 p.I368N mutant has a reduced protein half-life

**Fig. 5:**
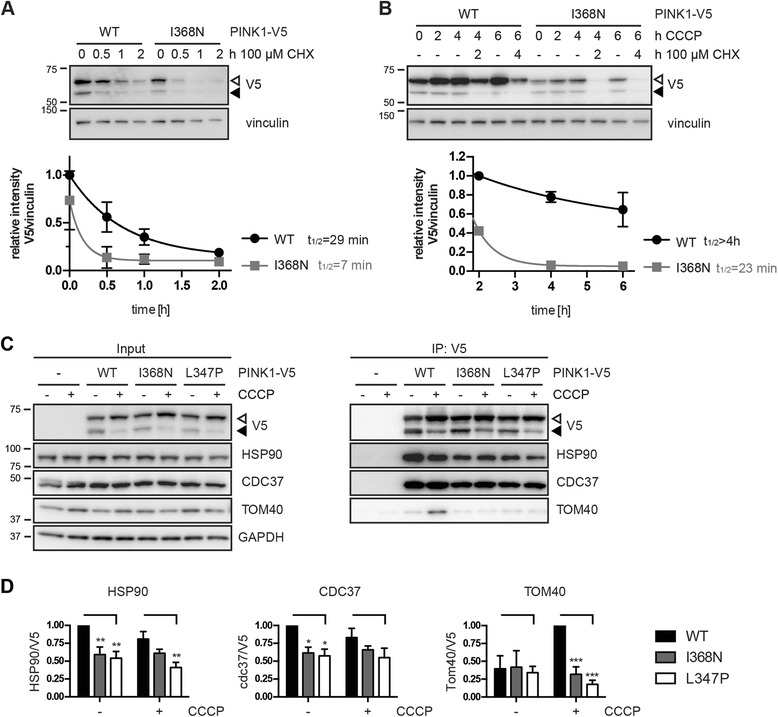
The half-life of the p.I368N mutant PINK1 protein is reduced. **a**-**b** HeLa cells were simultaneously transfected with PINK1 siRNA and DNA constructs encoding for PINK1-V5 WT or p.I368N mutant and incubated with 100 μg/ml cycloheximide (CHX) alone (**a**) or pretreated with 10 μM CCCP for 2 h before incubation with cycloheximide (**b**). Cell lysates were analyzed by WB and V5/vinculin ratios were normalized to PINK1-V5 WT. Shown is the mean ± SEM from three independent experiments. Half-life (t_1/2_) of PINK1-V5 proteins was determined by curve fitting (one phase exponential decay). **c** Co-immunoprecipitation (IP) of the HSP90/CDC37 chaperone complex and the import channel of the mitochondrial outer membrane TOM40 with PINK1-V5. HeLa cells were transfected with PINK1-V5 WT, p.I368N or another PD-mutant PINK1 p.L347P as a control and left untreated or treated with 10 μM CCCP for 2 h. PINK1-V5 protein complexes were immunoprecipitated from cell lysates using V5 antibody conjugated to agarose. Representative WB is shown for input and IP samples probed with antibodies against V5 (PINK1), HSP90, CDC37, and TOM40. GAPDH served as a loading control. **d** Quantification of the co-immunoprecipitated proteins as performed in (**c**) is shown as the protein/V5 ratio. Data represent mean ± SEM from six independent experiments. Statistical significance was assessed by two-way ANOVA with Tukey’s post hoc; ***, *p* < 0.0005; **, *p* < 0.005; *, *p* < 0.05

To better characterize the instability of PINK1 p.I368N over time, we performed experiments in the presence of a protein synthesis inhibitor. PINK1 siRNA knockdown HeLa cells were transfected with only 25% of the recommended DNA amounts (0.25 μg) to ensure low expression of PINK1-V5 close to endogenous levels. Cells were left untreated or treated with cycloheximide (CHX) and the decay of full-length PINK1 protein was monitored in the absence or presence of CCCP, respectively (Fig. 5a and b). Under basal conditions, without mitochondrial damage, the half-life of PINK1-V5 WT was approximately 29 min, while PINK1 p.I368N was stable for only about 7 min (Fig. 5a). Mitochondrial damage dramatically increased the half-life of PINK1-V5 WT (>4 h) and slightly increased also the stability of the p.I368N mutant to 23 min (Fig. 5b). The calculated half-life for PINK1 WT was in good agreement with a previous report [69]. Together this confirms that PINK1 p.I368N is normally expressed, similar to PINK1 WT, but is destabilized on the protein level, in particular during mitochondrial stress.

### Full-length PINK1 p.I368N is not stabilized on the OMM/by the HSP90/CDC37 co-chaperone complex

To elucidate the reasons for the destabilization of the p.I368N mutation, we investigated key protein interactions of PINK1 by co-immunoprecipitation (Fig. 5c and d). We focused on the HSP90/CDC37 chaperone complex that supports folding of several kinases in the cytosol including PINK1 [70]. The PD-associated PINK1 mutation p.L347P had been reported before as unstable due to its of lower binding affinity for HSP90/CDC37 [66, 71] and thus was included as a control. While PINK1-V5 WT co-immunoprecipitated substantial amounts of the two chaperones with and without CCCP treatment, both proteins were significantly reduced in immunoprecipitates of PINK1-V5 p.I368N and p.L347P, respectively. In addition, we examined the interaction of PINK1 with the integral OMM protein TOM40 that forms the channel of the protein import complex [72]. It is known that full-length PINK1 accumulates into a higher molecular weight protein complex with the import machinery [10, 73], which regulates its insertion into the OMM, stability and enzymatic function towards PARKIN activation. In line with this, PINK1-V5 WT was found to interact with TOM40 specifically upon CCCP treatment, while the PINK1 mutations p.I368N or p.L347P failed, despite comparable amounts of immunoprecipitated PINK1-V5. Together, these data suggest that the stability of the PINK1 p.I368N mutation is altered through reduced interactions with stabilizing chaperone and the mitochondrial protein import complex.

### The PINK1 p.I368N mutant affects kinase activity

**Fig. 6:**
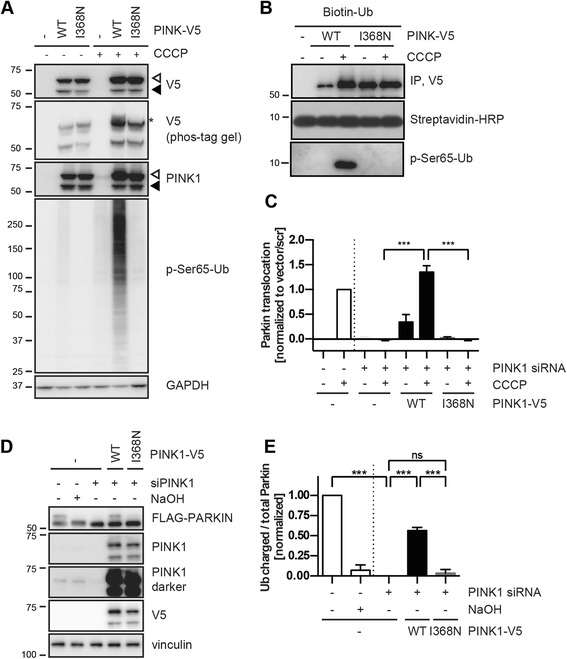
PINK1 p.I368N is a kinase dead mutant that fails to activate PARKIN and the mitochondrial quality control. **a** HeLa cells were simultaneously transfected with PINK1 siRNA and V5 empty vector, PINK1 WT or p.I368N mutant. Cells were left untreated or incubated with 10 μM CCCP for 4 h. Cell lysates were analyzed by WB, probed with anti-V5, PINK1 and p-Ser65-Ub antibodies. GAPDH was used as a loading control. Auto-phosphorylated PINK1 (anti-V5) (asterisk) was detected on phos-tag gels only in lysates from PINK1-V5 WT transfected, CCCP treated cells. In PINK1-V5 p.I368N transfected cells, CCCP-induced phosphorylation of Ub was largely abolished. **b** IP of PINK1-V5 using V5 antibody followed by *in vitro* kinase assay. HeLa cells were transfected with the V5 empty vector, PINK1-V5 WT or p.I368N and treated with or without CCCP for 2 h. Washed immunoprecipitates were incubated with N-terminally biotinylated mono-Ub and 500 μM ATP in phosphorylation buffer at 37 °C for 24 h. Total and phosphorylated Ub were analyzed by WB using streptavidin-HRP and p-Ser65-Ub antibody, respectively. **c** PARKIN translocation to damaged mitochondria measured by HCI. HeLa cells stably overexpressing GFP-tagged PARKIN were simultaneously transfected with PINK1 (or control) siRNA and with V5 empty vector, PINK1-V5 WT or p.I368N mutant, as indicated, and with mCherry as a selection marker. Cells were left untreated or treated with CCCP for 2 h and GFP-PARKIN translocation was measured in mCherry-positive cells. Data represents the mean of two independent experiments run with each six replicate wells. Statistical significance was assessed by two-way ANOVA with Tukey’s post hoc; **, *p* < 0.005; ***, *p* < 0.0005). **d** Ub-charging of PARKIN C431S as a measure of its enzymatic activation. HeLa cells stably expressing 3xFLAG-tagged PARKIN C431S were pretreated with control or *PINK*1 siRNA and then transfected with V5 empty vector, PINK1-V5 WT or p.I368N. After treatment with CCCP for 4 h, cell lysates were analyzed by WB with the indicated antibodies. PARKIN C431S traps Ub in an oxyester-bond (resulting in an 8 kDa band shift) that is cleavable by NaOH treatment. **e** Densitometric analysis of Ub-charged 3xFLAG-PARKIN that was normalized to total PARKIN levels. Error bars indicate mean ± SEM from three independent experiments (one-way ANOVA with Tukey’s post hoc; ***, *p* < 0.0005; ns, not significant)

To investigate kinase activity of the PINK1 mutation independent of protein instability, HeLa cells were transfected with WT or p.I368N mutant using standard methods (1 μg DNA). At equal expression levels, only PINK1 WT was auto-phosphorylated upon mitochondrial depolarization as shown by separation and detection on phos-tag gel (Fig. 6a). Accordingly, p-Ser65-Ub levels were only increased in HeLa cells transfected with PINK1 WT, but not in cells expressing the p.I368N mutant or the empty vector, also when endogenous PINK1 WT was silenced (Additional file 6: Figure S2B). To directly confirm lack of kinase activity of PINK1 p.I368N, we performed *in vitro* kinase assays with recombinant Ub. PINK1-V5 WT and p.I368N were immunoprecipitated from overexpressing cells that had been treated with or without CCCP and samples were incubated with biotinylated mono-Ub as a substrate. Phosphorylated Ub was detected exclusively in presence of PINK1 WT obtained from CCCP-treated cells (Fig. 6b).

To confirm a biological downstream effect, we analyzed translocation of GFP-PARKIN to damaged mitochondria in stable HeLa cells by HCI [61]. As expected, siRNA-mediated knockdown of endogenous PINK1 fully abrogated PARKIN translocation to damaged mitochondria (Fig. 6c), while exogenous expression of PINK1 WT restored it but the PINK1 p.I368N mutant failed. To biochemically verify failure to activate PARKIN, PINK1 WT or p.I368N mutant were transiently transfected into HeLa cells stably expressing an active-site mutant of PARKIN. PARKIN C431S traps Ub in an oxyester bond (8 kDa band shift) and facilitates the analysis of the E3 ligase’s Ub-charging as a read-out for its activation [22]. Consistently, significant PARKIN C431S ~ Ub levels were only observed upon re-transfection of cells with PINK1 WT but not with the p.I368N mutant or an empty vector (Fig. 6d, e). Altogether, these data confirm the lack of p.I368N kinase activity even at protein levels comparable to PINK1 WT, and thus its inability to activate PARKIN and the protective mitochondrial quality control.

## Discussion

While EOPD-associated missense mutations in PINK1 and PARKIN typically result in biological loss-of-function, their particular pathomechanisms may differ. Several studies have highlighted specific molecular defects of individual missense variants along a sequential and complex-regulated mitochondrial quality control pathway [5, 22, 24]. In fact, detailed analyses of PARKIN variants have significantly contributed to a better understanding of the pathway itself, the molecular events and their sequence. This is of particular interest not only towards a complete understanding of the PINK1/PARKIN pathway, but also towards a rationalized drug design in the future.

By now numerous missense mutations have been identified in PINK1 from both patients and controls [74]. However, the molecular pathomechanisms of PINK1 mutations have not been well investigated, in particular not on the endogenous level in patients’ cells. We have just recently shown that various mutations disrupt the enzymatic and biological functions of PINK1 at different steps [24, 25]. In addition to simple loss-of-function, specific PINK1 mutations can also exert a partial, dominant-negative function, simply through formation of a heterodimer with PINK1 WT [26]. This results in a significantly increased risk for PD even in heterozygotes. In this study, we revealed two additional molecular mechanisms that can lead to loss of PINK1 function and to increased disease risk.

PINK1 p.I368N was normally expressed, imported into and processed in mitochondria under non-stress conditions, in contrast to PINK1 WT, however, stabilization of the full-length mutant protein on the OMM was strongly impaired. Mitochondrial depolarization combined with proteasome inhibition stabilized similar levels of the PARL-cleaved forms of both PINK1 WT and p.I368N mutant. Yet, proteasome inhibition blocks progression of mitochondrial quality control and N-terminally processed PINK1 itself, which tends to aggregate in the insoluble fraction [7, 66, 67], and has been suggested to block PARKIN translocation from the cytosol [75]. To prevent potentially confounding effects, we employed the PINK1 p.F104A mutation that blocks degradation of the PARL-cleaved form through the N-end rule pathway even without proteasome inhibition [7]. Interestingly, a p.F104A/p.I368N double mutant showed slightly reduced levels of full-length and slightly, but significantly, enhanced levels of cleaved PINK1, compared to the p.F104A mutation alone. This could indicate an overall enhanced and potentially continuous import of PINK1 p.I368N that is coupled to PARL cleavage and degradation by the UPS in the cytosol. It has previously been suggested that the N-terminus of PINK1 is still imported into the inter-membrane space even after depolarization [76].

In addition to this, we found significantly reduced interactions of PINK1 p.I368N with the cytosolic chaperones HSP90 and CDC37. This chaperone system promotes folding of many kinases and has been shown to regulate stability and subcellular distribution of PINK1 [69, 70, 77, 78]. Mutations such as p.L347P that showed reduced binding to HSP90/CDC37 [71, 79] were suggested before to be subject to misfolding and increased turnover through either the Ub/proteasome system or another protease [66]. Similarly, protein half-life of full-length PINK1 p.I368N was reduced, both before and after mitochondrial stress. While we cannot formally exclude aberrant cleavage of the PINK1 p.I368N mutant in either compartment, a particular instability of the protein in the cytosolic vs. mitochondrial milieu was obvious.

It has been suggested that import of PINK1 into mitochondria is directly regulated by binding to HSP90 and CDC37. The chaperone complex not only assists in folding, stability, and thus kinase activity of PINK1 [66, 69], but also contributes to retention and accumulation of PINK1 on the OMM. As such, a reduced interaction would increase PINK1 inside mitochondria [79], which in turn would provide more substrate to MPP in the matrix and PARL in the IMM. Of note, loss of those proteases resulted in OMM accumulation of endogenous, full-length PINK1, which induced mitophagy even in the absence of membrane depolarization [8, 9, 76].

In this regard it is important to mention that PINK1 WT interacted with the mitochondrial import channel TOM40 specifically upon CCCP treatment, but both of the mutations that had reduced HSP90/CDC37 binding, failed. It is known that upon mitochondrial depolarization PINK1 auto-phosphorylates [73, 80] and dimerizes into a higher molecular weight protein complex together with the TOM machinery in the OMM [10, 12, 81]. However, it is unclear whether PINK1 then remains in the TOM complex or integrates laterally into the lipid phase of the OMM [9, 10, 76] once it has acquired its orientation to phosphorylate Ub and PARKIN.

In the structure of PINK1, Ile368 is positioned next to the catalytic loop residues 360-367 of the kinase domain. The catalytic loop contains a conserved catalytic base residue Asp362 that is essential for transfer of phosphate from ATP to the substrate [77]. Based on our structural analyses, we conclude that the mutation p.I368N induces local perturbations such that Asp362 cannot adopt the appropriate orientation needed for phosphate transfer. In addition, the de-formed ATP-binding pocket may de-stabilize the interaction which could result in dissociation of ATP from PINK1. All of this together with altered Ub binding would additively decrease the kinase activity of PINK1 p.I368N. While recombinant human PINK1 is notoriously difficult to purify as an active protein in sufficient amounts, it will be important to validate our computational predictions by structural and biophysical comparisons of WT and mutant forms.

Nevertheless, similar defects might be expected to underlie the pathogenicity of other PD associated mutations that are nearby, such as p.N367S or p.L369P. Importantly, a recent study had identified the ATP-analog kinetin to rescue a kinase-defective mutant PINK1 p.G309D [82]. Future rationalized drug design that is based on a detailed understanding of the pathomechanisms should furnish more such small molecules. In addition to active site binders, the complex and as of yet poorly understood life cycle of PINK1 that includes its import, cleavage and re-routing between both mitochondrial membranes might reveal further allosteric sites or strategies to enhance activity and/or retention/stabilization on the OMM.

Some studies have already raised a note of caution as massive overexpression of PINK1 can result in its stabilization on the OMM and subsequent PARKIN recruitment to even healthy mitochondria [11, 16, 68, 80]. In line, our data indicate that subtle defects seen with physiological levels of PINK1 can be masked or overrun by overexpression. Here, we showed that full-length PINK1 p.I368N is unstable on the OMM under endogenous conditions, but this effect was not seen in the overexpression situation. In addition, in a recent study we have found a partial loss-of-function for the PINK1 p.G411S mutation under endogenous conditions [26], which was missed in an earlier overexpression study [68].

In summary, we have shown that the PINK1 p.I368N mutation affects protein stabilization specifically on the OMM and results in complete loss of kinase activity towards Ub likely caused by distortion of the ATP-binding site. Together with earlier studies, this highlights the presence of several distinct pathomechanisms that inactivate PINK1 and emphasizes at novel therapeutic avenues that could be pursued in a structure-function based drug design.

## Conclusions

Mutations in PINK1 and PARKIN are the most common forms of EOPD. Both proteins play critical roles in stress-induced mitochondrial quality control that protects cells from death. In this study, we examined the PINK1 p.I368N mutation on the clinical and genetic as well as the structural and functional level. We identified two distinct molecular mechanisms that can lead to inactivation of PINK1 function and thus result in loss of neuroprotection. Our research highlights future avenues for drug design that are based on an understanding of the pathogenic events underlying loss of PINK1 activity.

## Additional files

Additional file 1: Table S1. Clinical features of PD patients homozygous for the PINK1 p.I368N mutation are summarized. Abbreviations: RBD: REM sleep behavior disorder; MMSE: Mini Mental State Examination; DBS: deep brain stimulation. (DOCX 16 kb)

Additional file 4: Table S2. FoldX measurement for mutational energy effect on stability of binding pocket. Using the FoldX algorithm, which compares the WT structure with the mutant, calculations on WT and mutant at t = 0 and after 100 ns were performed. While WT is nearly zero over that time interval, the N368 mutant increases energy (∆∆G) by 1.5–2 kcal/mol*Å^2^. This modest increase supports that the mutation distorts the local region via an increase of Gibb’s free energy. (DOCX 16 kb)

Additional file 5: Figure S1. Reduced full-length PINK1 levels in p.I368N mutant fibroblasts upon treatment with valinomycin. (A) Representative confocal IF images of control and two PINK1 p.I368N fibroblasts showing mitochondrial localization but greatly reduced levels of the mutant protein. Cells were left untreated (-) or treated with 10 μM CCCP for 6 days (+) and stained with antibodies against PINK1 (green) and TOM20 (mitochondria, red). Nuclei were counterstained with Hoechst (blue). Scale bars represent 10 μm. (B) Subcellular fractionation of WT control and PINK1 p.I368N fibroblasts treated with or without 1 μM valinomycin for 24 h. Despite different protein levels, both WT and p.I368N mutant full-length PINK1 localized to the mitochondrial fraction. A shift of PARKIN into higher molecular weights species indicative of PINK1-dependent activation was not observed in lysates from PINK1 p.I368N mutant cells. Purity of the mitochondrial and cytosolic fractions was determined using antibodies recognizing TOM20 and p38 MAPK, respectively. PNS denotes post-nuclear supernatant. (C) Control and two PINK1 p.I368N fibroblasts were treated with 1 μM valinomycin for 0, 2, 4 or 8 h and total lysates were analyzed by WB with indicated antibodies. GAPDH served as a loading control. Similar to CCCP treatment (Fig. 3b), rapid stabilization of full-length PINK1 along with an increase of p-Ser65-Ub levels was observed in control cells, but not in PINK1 p.I368N mutant fibroblasts. (D) Representative Images obtained with a 20x magnification on the BD pathway 855 system. Control fibroblasts and PINK1 I368N cells were seeded in 96-well imaging plates and treated with valinomycin as indicated. Cells were fixed and stained with p-S65-Ub antibodies (green) and Hoechst (blue). Scale bars indicate 10 μm. (TIF 13839 kb)

Additional file 6 **Figure S2**. Expression of PINK1-V5 WT and p.I368N mutation at different levels in HeLa cells. (A) HeLa cells were transfected with PINK1 siRNA and with different amounts (0.5 or 1 μg per well of a 12-well plate) of V5 empty vector, PINK1-V5 WT or p.I368N, as indicated. Cells were left untreated or incubated with 10 μM CCCP for 4 h and lysates were analyzed on WB with anti-V5 and PINK1 antibodies. GAPDH was used as a loading control. V5/GAPDH ratios were determined by densitometry and normalized to values of CCCP treated PINK1 WT samples transfected with 1 μg of DNA. (B) Denitometric analysis of data from (A). Shown is the mean ± SEM from four independent experiments. Statistical significance was assessed by two-way ANOVA with Tukey’s post hoc; *, *p* < 0.05; ***, *p* < 0.0005; ns, not significant. (C) HeLa cells were transfected with control or PINK1 siRNA and with V5 empty vector, PINK1-V5 WT or p.I368N. Cells were treated with CCCP for 4 h and proteins were extracted and analyzed by WB using the indicated antibodies. p-Ser65-Ub signal was detected only in PINK1-V5 WT but not in p.I368N transfected cells upon mitochondrial stress despite similar PINK1 proteins levels. (TIF 3542 kb)

## Abbreviations

CCCP: Carbonyl cyanide *m*-chlorophenyl hydrazone
CHX: Cycloheximide
EOPD: Early-onset Parkinson’s disease
HCI: High content imaging
IF: Immunofluorescence
IMM: Inner mitochondrial membrane
IP: Immunoprecipitation
MPP: Matrix processing peptidase
MSD: Molecular dynamics simulation
MTS: Mitochondrial targeting sequence
OMM: Outer mitochondrial membrane
PARL: Presenilin-associated rhomboid-like protein
PD: Parkinson’s disease
PINK1: PTEN-induced putative kinase 1
p-Ser65-Ub: PINK1 phosphorylated ubiquitin (Serine-65)
qRT-PCR: Quantitative reverse transcriptase polymerase chain reaction
RMSD: Root mean square deviation
RMSF: Root mean square fluctuation
TIM: Translocase of the inner mitochondrial membrane
TMD: Transmembrane domain
TOM: Translocase of the outer mitochondrial membrane
Ub: Ubiquitin
UPS: Ubiquitin-proteasome system
WB: Western blot
WT: Wild type
