# Supplementary material for: The PINK1 p.I368N mutation affects protein stability and ubiquitin kinase activity
Source: Mol Neurodegener. 2017 Apr 24;12:32. doi: 10.1186/s13024-017-0174-z (PMC5404317; doi:10.1186/s13024-017-0174-z)
Supplement: Supplementary file 3 — Clinical features of PD patients homozygous for the PINK1 p.I368N mutation are summarized. Abbreviations: RBD: REM sleep behavior disorder; MMSE: Mini Mental State Examination; DBS: deep brain stimulation. (DOCX 16 kb) [file 13024_2017_174_MOESM1_ESM.docx]

**Additional file 1: Table S1**

| **general** | **PINK1 patient** | **I368N #1** | **I368N #2** |
| --- | --- | --- | --- |
| **information** |  |  |  |
|  | age of onset | 28 | 33 |
| **phenotype** | laterality | + | + |
|  | bradykinesia | + | + |
|  | activation tremor | - | - |
|  | resting tremor | + | + |
|  | postural instability | + | - |
|  | rigidity | + | + |
|  | gait difficulties | + | + |
|  | gaze palsy | - | - |
|  | cerebellar sign | - | - |
|  | oculogyric crisis | - | - |
|  | RBD | - | - |
|  | depression/ anxiety | - | - |
|  | hallucination | - | - |
|  | dysautonomia | + | + |
|  | significant cognitive impairment or dementia | - | - |
|  | response to anti-parkinsonism therapy | + | + |
| **past history** | stroke/stepwise deterioration | - | - |
|  | head injury | - | - |
|  | loss of consciousness | - | - |
|  | encephalitis | - | - |
|  | neuroleptic treatment at time of symptom onset | - | - |
| **other** | Hohn & Yahl | 3 | 3 |
|  | MMSE | 28 | 29 |
|  | handedness | right | right |
|  | DBS | - | - |
