# Supplementary material for: The PINK1 p.I368N mutation affects protein stability and ubiquitin kinase activity
Source: Mol Neurodegener. 2017 Apr 24;12:32. doi: 10.1186/s13024-017-0174-z (PMC5404317; doi:10.1186/s13024-017-0174-z)
Supplement: Supplementary file 4 — FoldX measurement for mutational energy effect on stability of binding pocket. Using the FoldX algorithm, which compares the WT structure with the mutant, calculations on WT and mutant at t = 0 and after 100 ns were performed. While WT is nearly zero over that time interval, the N368 mutant increases energy (∆∆G) by 1.5–2 kcal/mol*Å2. This modest increase supports that the mutation distorts the local region via an increase of Gibb’s free energy. (DOCX 16 kb) [file 13024_2017_174_MOESM4_ESM.docx]

Additional file 4: Table S2

| WT residue (normalized) | | FOLD X (kcal/mol*Å^2^) | | |
| --- | --- | --- | --- | --- |
| I368 (t=0) | I368  (t>100 ns) | N368 mutant (t=0)$\Delta\Delta$G | N368 mutant (t>100 ns) $\Delta\Delta$G | Overall Stability |
| 0 | <0.3 | 1.37 | 1.56 – 2.1 | Less stable pocket |
