# Supplementary figures and images for: The PINK1 p.I368N mutation affects protein stability and ubiquitin kinase activity
Source: Mol Neurodegener. 2017 Apr 24;12:32. doi: 10.1186/s13024-017-0174-z (PMC5404317; doi:10.1186/s13024-017-0174-z)

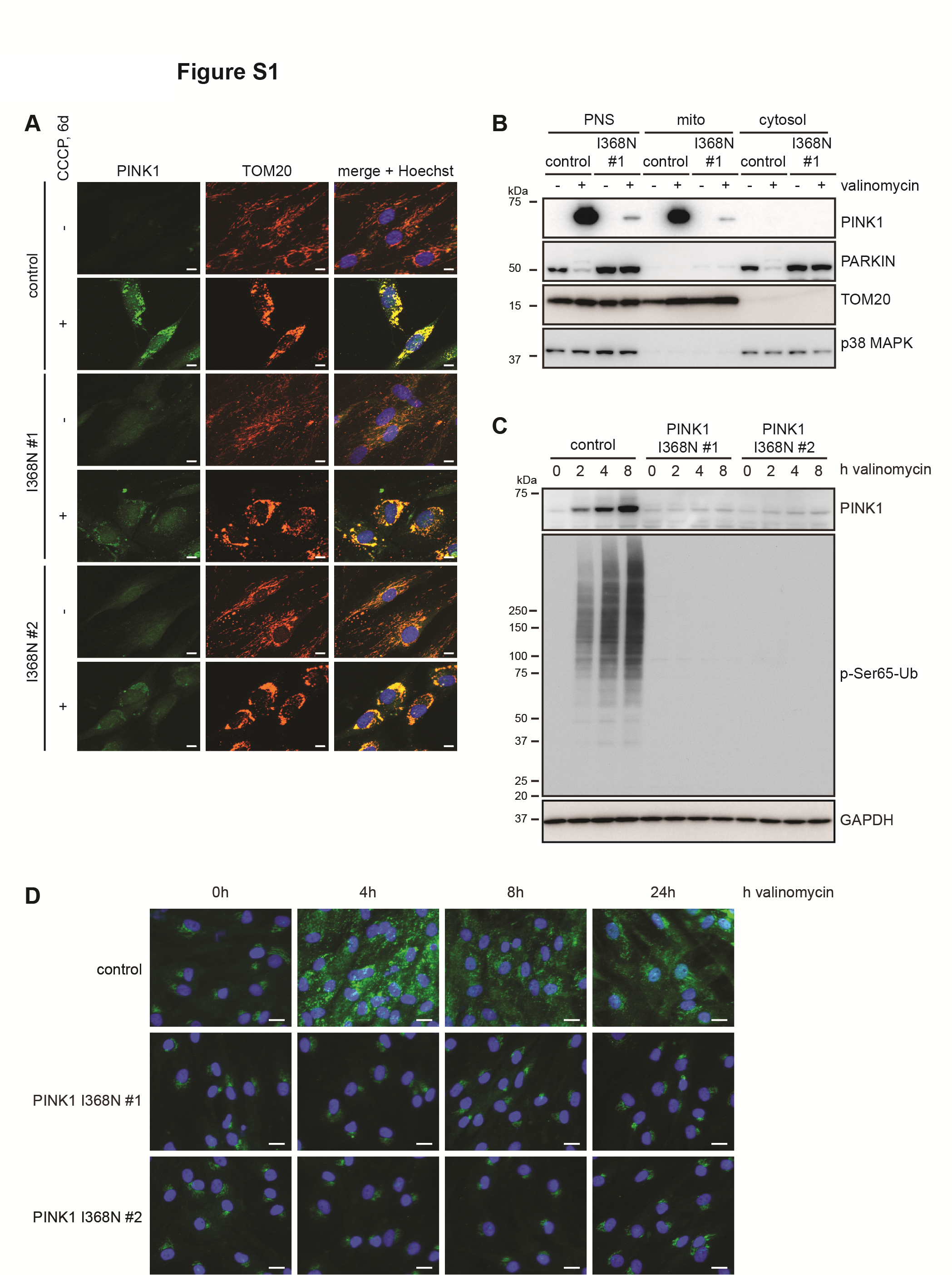

Supplement: Supplementary file 5 — Reduced full-length PINK1 levels in p.I368N mutant fibroblasts upon treatment with valinomycin. (A) Representative confocal IF images of control and two PINK1 p.I368N fibroblasts showing mitochondrial localization but greatly reduced levels of the mutant protein. Cells were left untreated (-) or treated with 10 μM CCCP for 6 days (+) and stained with antibodies against PINK1 (green) and TOM20 (mitochondria, red). Nuclei were counterstained with Hoechst (blue). Scale bars represent 10 μm. (B) Subcellular fractionation of WT control and PINK1 p.I368N fibroblasts treated with or without 1 μM valinomycin for 24 h. Despite different protein levels, both WT and p.I368N mutant full-length PINK1 localized to the mitochondrial fraction. A shift of PARKIN into higher molecular weights species indicative of PINK1-dependent activation was not observed in lysates from PINK1 p.I368N mutant cells. Purity of the mitochondrial and cytosolic fractions was determined using antibodies recognizing TOM20 and p38 MAPK, respectively. PNS denotes post-nuclear supernatant. (C) Control and two PINK1 p.I368N fibroblasts were treated with 1 μM valinomycin for 0, 2, 4 or 8 h and total lysates were analyzed by WB with indicated antibodies. GAPDH served as a loading control. Similar to CCCP treatment (Fig. 3b), rapid stabilization of full-length PINK1 along with an increase of p-Ser65-Ub levels was observed in control cells, but not in PINK1 p.I368N mutant fibroblasts. (D) Representative Images obtained with a 20x magnification on the BD pathway 855 system. Control fibroblasts and PINK1 I368N cells were seeded in 96-well imaging plates and treated with valinomycin as indicated. Cells were fixed and stained with p-S65-Ub antibodies (green) and Hoechst (blue). Scale bars indicate 10 μm. (TIF 13839 kb) [file 13024_2017_174_MOESM5_ESM.tif]

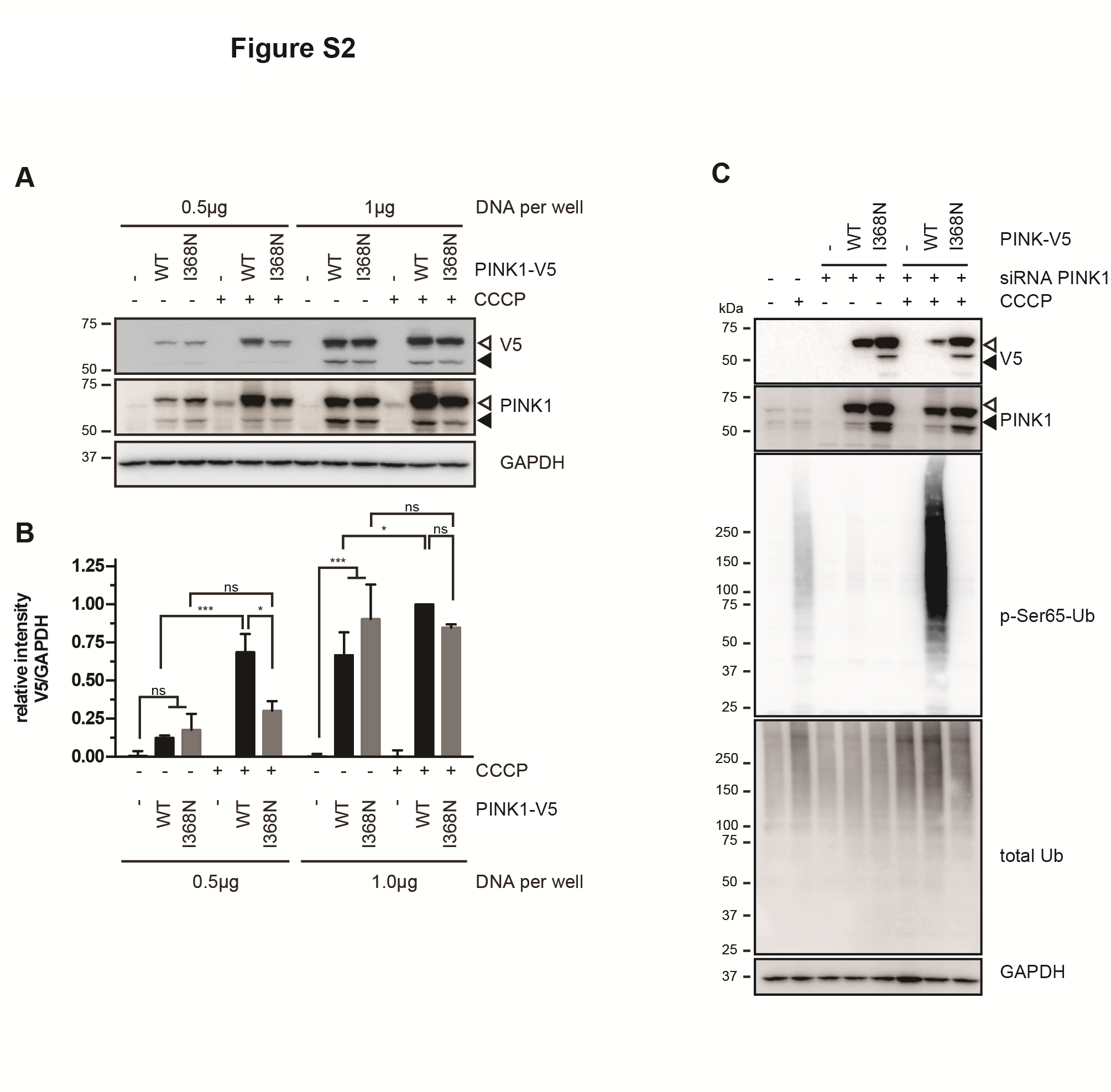

Supplement: Supplementary file 6 — Figure S2. Expression of PINK1-V5 WT and p.I368N mutation at different levels in HeLa cells. (A) HeLa cells were transfected with PINK1 siRNA and with different amounts (0.5 or 1 μg per well of a 12-well plate) of V5 empty vector, PINK1-V5 WT or p.I368N, as indicated. Cells were left untreated or incubated with 10 μM CCCP for 4 h and lysates were analyzed on WB with anti-V5 and PINK1 antibodies. GAPDH was used as a loading control. V5/GAPDH ratios were determined by densitometry and normalized to values of CCCP treated PINK1 WT samples transfected with 1 μg of DNA. (B) Denitometric analysis of data from (A). Shown is the mean ± SEM from four independent experiments. Statistical significance was assessed by two-way ANOVA with Tukey’s post hoc; *, p < 0.05; ***, p < 0.0005; ns, not significant. (C) HeLa cells were transfected with control or PINK1 siRNA and with V5 empty vector, PINK1-V5 WT or p.I368N. Cells were treated with CCCP for 4 h and proteins were extracted and analyzed by WB using the indicated antibodies. p-Ser65-Ub signal was detected only in PINK1-V5 WT but not in p.I368N transfected cells upon mitochondrial stress despite similar PINK1 proteins levels. (TIF 3542 kb) [file 13024_2017_174_MOESM6_ESM.tif]
